# Molnupiravir for the treatment of COVID-19 in immunocompromised participants: efficacy, safety, and virology results from the phase 3 randomized, placebo-controlled MOVe-OUT trial

**DOI:** 10.1007/s15010-022-01959-9

**Published:** 2023-01-17

**Authors:** Matthew G. Johnson, Julie M. Strizki, Michelle L. Brown, Hong Wan, Hala H. Shamsuddin, Moti Ramgopal, Diana F. Florescu, Pierre Delobel, Ilsiyar Khaertynova, José F. Flores, Leon F. Fouche, Shan-Chwen Chang, Angela Williams-Diaz, Jiejun Du, Jay A. Grobler, Amanda Paschke, Carisa De Anda

**Affiliations:** 1 Merck & Co., Inc., Rahway, NJ USA; 2 Midway Immunology and Research Center, Fort Pierce, FL USA; 3 University of Nebraska Medical Center, Omaha, NE USA; 4 Université Toulouse III Paul Sabatier, CHU de Toulouse, Toulouse, France; 5 Republican Clinical Infectious Diseases Hospital n.a. A.F. Agafonov, Kazan, Russian Federation; 6 Clinica Privada Dr. José Francisco Flores López, Guatemala, Guatemala; 7 Limpopo Clinical Research Initiative, Thabazimbi, South Africa; 8 National Taiwan University Hospital, Taipei, Taiwan

**Keywords:** COVID-19, Immunocompromised, Molnupiravir, Treatment, Virology

## Abstract

**Purpose:**

Immunocompromised patients have a potentially increased risk for progression to severe COVID-19 and prolonged replication of SARS-CoV-2. This post hoc analysis examined outcomes among immunocompromised participants in the MOVe-OUT trial.

**Methods:**

In phase 3 of MOVe-OUT, non-hospitalized at-risk adults with mild-to-moderate COVID-19 were randomized to receive molnupiravir 800 mg or placebo twice daily for 5 days. Immunocompromised participants were identified based on prior/concomitant medications and/or medical history. All-cause hospitalization/death, adverse events, SARS-CoV-2 titers, infectivity, and RNA sequences were compared between immunocompromised participants who received molnupiravir or placebo and with non-immunocompromised participants.

**Results:**

Fifty-five of 1408 participants were considered immunocompromised. Compared to placebo, fewer molnupiravir-treated immunocompromised participants were hospitalized/died through Day 29 (22.6% [7/31] vs. 8.3% [2/24]), with fewer adverse events (45.2% [14/31] vs. 25.0% [6/24]). A larger mean change from baseline in SARS-CoV-2 RNA was observed with molnupiravir compared to placebo in non-immunocompromised participants (least squares mean [LSM] difference Day 5:  – 0.31, 95% confidence interval [CI]  – 0.47 to  – 0.15), while the mean change was comparable between treatment groups in immunocompromised participants (LSM difference Day 5: 0.23, 95% CI  – 0.71 to 1.17). Molnupiravir treatment was associated with increased clearance of infectious virus. Increased errors in viral nucleotide sequences in post-baseline samples compared to placebo support molnupiravir’s mechanism of action and were not associated with observation of novel treatment-emergent amino acid substitutions in immunocompromised participants.

**Conclusion:**

Although the study population was small, these data suggest that molnupiravir treatment for mild-to-moderate COVID-19 in non-hospitalized immunocompromised adults is efficacious and safe and quickly reduces infectious SARS-CoV-2.

**ClinicalTrials.gov Registration Number:**

NCT04575597.

**Supplementary Information:**

The online version contains supplementary material available at 10.1007/s15010-022-01959-9.

## Introduction

Immunocompromised individuals infected with severe acute respiratory syndrome coronavirus 2 (SARS-CoV-2) are more likely to progress to severe coronavirus disease 2019 (COVID-19) and have poor outcomes [1–6]. Additionally, immunocompromised individuals, such as hematopoietic stem cell and solid organ transplant recipients, and people living with HIV (PLWH) with CD4 + T lymphocyte counts below 200 cells/mm^3^, may have prolonged viral shedding compared with non-immunocompromised individuals [7–11]. Factors associated with immunosuppression that negatively impact COVID-19 outcomes and delay viral clearance include CD4 + T lymphocyte count below 200 cells/mm^3^, cancer type, allograft type, time from transplant, and type and dose of chemotherapy, immunotherapy, or maintenance immunosuppression after induction [3, 4, 6–11].

Molnupiravir is an oral, small-molecule ribonucleoside prodrug of β-D-N4-hydroxycytidine (NHC) which has potent activity against SARS-CoV-2, including all variants of concern to date [12–14]. NHC inhibits SARS-CoV-2 by introducing random nucleotide errors across the viral RNA genome leading to loss of viral infectivity [15, 16]. Preclinical in vivo and clinical phase 2 and 3 studies have confirmed that molnupiravir reduces viral infectivity [17–20], with no infectious virus detected on Day 5 (end-of-therapy, EOT) in participants who were positive for infectious virus at baseline and received molnupiravir 800 mg twice daily for 5 days for the treatment of mild-to-moderate COVID-19 [19, 20].

MOVe-OUT was a phase 2/3 randomized, double-blind, placebo-controlled trial that evaluated molnupiravir for the treatment of non-hospitalized adults with mild-to-moderate COVID-19 and risk factors for progression to severe disease. In the interim analysis for the phase 3 component of MOVe-OUT (enrollment May 6 through August 5, 2021), molnupiravir was superior to placebo in reducing the risk for all-cause hospitalization or death by Day 29 (7.3% vs. 14.1%; difference,  – 6.8%; 95% CI,  – 11.3 to  – 2.4). In the final analysis including all randomized participants (enrollment May 6 through October 2, 2021), molnupiravir demonstrated a reduction in the risk for all-cause hospitalization or death (6.8% vs. 9.7%; difference,  – 3.0%; 95% CI,  – 5.9 to  – 0.1), with an 89% relative risk reduction in all-cause mortality compared with placebo [21].

This post hoc analysis explored virologic data, as well as clinical efficacy and safety, in the cohort of immunocompromised participants from the phase 3 component of MOVe-OUT. We compared all-cause hospitalization or death and adverse events along with SARS-CoV-2 viral RNA, viral infectivity, viral RNA nucleotide error rate, treatment-emergent amino acid changes, and anti-SARS-CoV-2 antibody status among immunocompromised and non-immunocompromised participants treated with molnupiravir versus placebo.

## Methods

### Design overview, setting, and participants

Non-hospitalized adults ≥ 18 years old with laboratory-confirmed mild-to-moderate COVID-19 and at least one risk factor for progression to severe disease were included in the phase 3 component of MOVe-OUT (ClinicalTrials.gov NCT04575597). Participants were randomized within 5 days of onset of COVID-19 signs or symptoms and received molnupiravir 800 mg every 12 h or matching placebo for 5 days. Nasopharyngeal swabs and blood samples were collected at baseline on Day 1 and on Days 3, 5, 10, 15, and 29. MOVe-OUT was conducted in accordance with local and/or national regulations (including all applicable data protection laws and regulations), International Council for Harmonization-Good Clinical Practice, and the ethical principles that have their origin in the Declaration of Helsinki regarding independent ethics committee review, informed consent, and the protection of human participants in biomedical research. The full details of the phase 3 component of MOVe-OUT, along with the study protocol, are reported in Jayk Bernal, A. et al. [21].

Immunocompromised participants were identified based on a post hoc review of medical history and/or prior and concomitant use of systemic corticosteroids or immunosuppressant medications, as classified in H02AB and L04 of the World Health Organization Anatomical Therapeutic Chemical Classification System code. The following criteria were applied to identify immunocompromised participants: prior use of systemic corticosteroids for ≤ 4 weeks prior to receipt of the first dose of study drug; prior and/or concomitant use of immunosuppressants for an underlying condition(s); and/or medical history of immunocompromising conditions, including HIV (PLWH receiving a stable antiretroviral regimen), hematopoietic stem cell or solid organ transplant recipient, and active cancer*.* Active cancer was identified at the discretion of the investigators. Participants with an absolute neutrophil count < 500 cells/mm^3^ or a recent HIV RNA > 50 copies/mL (regardless of CD4 + T lymphocyte count) or an acquired immunodeficiency syndrome-defining illness in the past 6 months were excluded. Participants who received immunosuppressants for the treatment of COVID-19 or had minor cancers not associated with immunosuppression or significant morbidity/mortality (e.g., basal cell carcinomas) were not considered immunocompromised for this analysis.

### Outcomes

All-cause hospitalization or death through Day 29 and virologic outcomes with molnupiravir and placebo in immunocompromised and non-immunocompromised participants were assessed in the modified intent-to-treat population (MITT, all randomized participants who received ≥ 1 dose of study drug and were not hospitalized prior to the first dose). Adverse events through Day 29 were summarized in the safety population (all randomized participants who received ≥ 1 dose of study drug). Virologic outcomes included mean change from baseline in quantitative SARS-CoV-2 RNA over time, proportion of participants with detectable infectious SARS-CoV-2 over time, SARS-CoV-2 nucleotide error rates at Day 5, treatment-emergent amino acid changes through Day 29, proportion of participants with SARS-CoV-2 nucleocapsid antibody positivity over time, and mean change from baseline in SARS-CoV-2 neutralizing antibody titers on Days 10 and 29.

### SARS-CoV-2 RNA

SARS-CoV-2 RNA titers from nasopharyngeal swabs on Days 1, 3, 5, 10, 15, and 29 were measured using a quantitative RT-PCR assay developed at Q^2^ Solutions (Morrisville, NC, USA). The assay reported SARS-CoV-2 RNA values between 500 to 500,000,000 copies/mL. To extend the dynamic range of the assay, samples resulting with titers above the upper limit of quantification (> 500,000,000 copies/mL) were diluted 100-fold and retested. RNA titers obtained from the retest of diluted sample were adjusted (100 ×) accordingly. If the calculated value of the diluted sample was lower than the original result, the higher value of the 2 determinations was used for analysis.

### SARS-CoV-2 infectivity

Nasopharyngeal specimens collected on Days 1, 3, 5, 10, 15, and 29 with viral RNA > 100,000 copies/mL were serially diluted in duplicate in serum free Eagle Minimum Essential Media and 100 µL of each dilution placed in a 24-well-plate containing > 90% confluent Vero E6 cells. Samples were incubated with cells for 60 min at 37°C and 5% CO_2_ before addition of 1 mL of overlay media. Cultures were incubated for 48 h, and the plaque-forming units (PFUs) were visualized by washing and staining with crystal violet solution for 30 min. Plaques were then manually counted, and the final infectious titer was calculated based on the dilution factor. A control reference standard sample with known PFU titer was assayed for every batch. The lower limit of quantification for this assay was determined as 200 PFU/mL. Samples with titer values below the lower limit were resulted as < 200 PFU/mL.

### Next-generation sequencing (NGS)

NGS analysis was performed on nasopharyngeal samples with RNA titers ≥ 600 copies/mL on Days 1 and 5 and on post-treatment nasopharyngeal samples with RNA titers ≥ 100,000 copies/mL on Days 10, 15, and 29. All NGS analyses were performed by Q^2^ Solutions (Morrisville, NC, USA). An Ion AmpliSeq SARS-CoV-2 Research panel (Thermo Fisher Scientific, Waltham, MA, USA) consisting of two primer pair pools that target 237 amplicons specific to the SARS-CoV-2 virus was used. This panel provides > 99% coverage of the SARS-CoV-2 genome. Reverse transcription of the viral RNA input was performed using the SuperScript VILO cDNA Synthesis kit. Library preparation was completed using the Ion AmpliSeq Library Kit Plus. After targeted PCR amplification using 19 cycles, libraries were partially digested, and barcode adapters were ligated to the fragment ends. The final library was quantified using the Ion Library TaqMan Quantitation kit to inform final library loading onto an Ion Torrent sequencer. A minimum of 250,000 reads were targeted for each sample. A positive control was included with each library batch to monitor reagent integrity, and a no template control was included to monitor for cross-contamination.

### Anti-SARS-CoV-2 nucleocapsid antibody assay

The presence of serum antibodies (IgM, IgG, and IgA) to the SARS-CoV-2 nucleocapsid protein were assessed at Days 1, 5, 10, and 29 using the Roche Elecsys® electrochemiluminescence immunoassay performed at a central laboratory (LabCorp, Inc.; Indianapolis, IN, USA).

### Anti-SARS-CoV-2 spike protein neutralizing antibody assay

Assessment for the presence and amount of anti-SARS-CoV-2 spike protein neutralizing antibody activity in serum on Day 1 and on Days 10 and 29 was performed at Monogram Biosciences (South San Francisco, CA, USA) using the SARS-CoV-2 PhenoSense® nAB Assay. Serially diluted (1:40–1:2124 dilutions) serum samples were added to HEK293/ACE2 target cells before infection with a pseudotyped luciferase reporter virus expressing the spike protein from the original Wu-1 strain. Serum neutralizing titers were calculated based on the serum dilution needed to inhibit 50% luciferase signal compared with negative control serum.

### Statistical analysis

Descriptive statistics were used to summarize efficacy, safety, and virologic data. For categorical variables, frequency and proportions were calculated using the number of participants with available data as the denominator. Differences in the proportion of participants who experienced hospitalization or death through Day 29 were estimated based on the Miettinen & Nurminen method [22]. Differences in least square means were used to characterize the changes from baseline in SARS-CoV-2 RNA titers over time.

### Role of the sponsor

The trial sponsor, Merck & Co., Inc., Rahway, NJ, USA, was involved in study design, data collection, data analysis, data interpretation, and writing of the report. All authors had access to the study data and final responsibility for the decision to submit for publication.

## Results

Among all participants in the MITT population, a total of 3.9% (55/1408) were identified as immunocompromised for this post hoc review. Two participants who received prior corticosteroids were not considered immunocompromised for this analysis (one molnupiravir-treated participant who received inhaled fluticasone/salmeterol and one-placebo treated participant who received oral betamethasone/dexchlorpheniramine and inhaled fluticasone/salmeterol. The most common immunocompromising condition in both treatment groups was active cancer (Table 1). Doses of prior corticosteroids and immunosuppressants were not decreased during the study. A larger proportion of immunocompromised participants were older, female, Black or African American, from Africa, and not obese compared with non-immunocompromised participants; fewer immunocompromised participants were positive for the spike protein neutralizing antibody at baseline than non-immunocompromised participants (Table 2).

**Table 1:** Immunocompromising conditions (safety population)

Immunocompromising conditions, *n*^a^	Molnupiravir (*n* = 24)	Placebo (*n* = 31)
Active cancer^b^	13	16
Stable, virologically suppressed HIV^c^	6	6
Immunosuppressant therapy^d^	6	8
Azathioprine	1	1
Etanercept	0	1
Golimumab	1	1
Lenalidomide	1	2
Methotrexate	1	3
Mycophenolate mofetil	2	2
Tacrolimus	2	2
Tocilizumab	0	1
Prior systemic corticosteroid therapy^e^	5	6
Transplant	3	2
Autologous bone marrow	1	0
Heart	2	1
Kidney	0	1

^a^Participants could have more than one immunocompromising condition

^b^Excluding minor cancers not associated with immunosuppression or significant morbidity/mortality. Active cancer was identified at the discretion of the investigators

^c^Excluding participants with a recent HIV RNA > 50 copies/mL (regardless of CD4 + T lymphocyte count) or an acquired immunodeficiency syndrome-defining illness in the past 6 months

^d^For an underlying medical condition (not for the treatment of COVID-19 or associated conditions)

^e^Included a variety of different corticosteroid types, dosing forms, dosages, and treatment durations (all for at least 4 weeks prior to the first dose of study drug)

**Table 2:** Baseline demographics and characteristics (MITT population)

Baseline demographic/characteristic	Immunocompromised		Non-immunocompromised	
	Molnupiravir (*n* = 24)	Placebo (*n* = 31)	Molnupiravir (*n* = 685)	Placebo (*n* = 668)
Female, *n* (%)	17 (70.8)	19 (61.3)	362 (52.8)	325 (48.7)
Median age (range)	49 (19–82)	48 (20–87)	42 (18–90)	43 (18–88)
Median time from symptom onset to randomization (range)	4 (2–5)	4 (1–5)	4 (1–5)	4 (1–5)
*Race, n* (%)				
White	7 (29.2)	11 (35.5)	390 (56.9)	394 (59.0)
Black or African American	6 (25.0)	6 (19.4)	33 (4.8)	28 (4.2)
American Indian or Alaska Native	2 (8.3)	2 (6.5)	58 (8.5)	41 (6.1)
Asian	0 (0.0)	1 (3.2)	25 (3.6)	21 (3.1)
Multiple	9 (37.5)	11 (35.5)	179 (26.1)	184 (27.5)
Hispanic or Latino, *n* (%)	11 (45.8)	16 (51.6)	340 (49.6)	330 (49.4)
*Region, n* (%)				
Latin America	11 (45.8)	16 (51.6)	318 (46.4)	305 (45.7)
Africa	9 (37.5)	4 (12.9)	81 (11.8)	80 (12.0)
Europe	4 (16.7)	7 (22.6)	225 (32.8)	226 (33.8)
North America	0 (0.0)	3 (9.7)	42 (6.1)	42 (6.3)
Asia Pacific	0 (0.0)	1 (3.2)	19 (2.8)	15 (2.2)
*Risk factors for severe COVID-19, n* (%)				
At least one risk factor	24 (100.0)	31 (100.0)	684 (99.9)	665 (99.6)
Age > 60 years	6 (25.0)	4 (12.9)	112 (16.4)	123 (18.4)
Active cancer	13 (54.2)	16 (51.6)	0 (0.0)	0 (0.0)
Chronic kidney disease	1 (4.2)	4 (12.9)	37 (5.4)	39 (5.8)
Chronic obstructive pulmonary disease	0 (0.0)	2 (6.5)	22 (3.2)	32 (4.8)
Body mass index ≥ 30	15 (62.5)	15 (48.4)	520 (75.9)	492 (73.7)
Serious heart condition	4 (16.7)	9 (29.0)	82 (12.0)	69 (10.3)
Diabetes mellitus	6 (25.0)	5 (16.1)	101 (14.7)	112 (16.8)
*COVID-19 severity, n* (%)				
Mild	13 (54.2)	13 (41.9)	382 (55.8)	363 (54.3)
Moderate	11 (45.8)	18 (58.1)	300 (43.8)	303 (45.4)
Severe	0 (0.0)	0 (0.0)	3 (0.4)	1 (0.1)
Unknown^a^	0 (0.0)	0 (0.0)	0 (0.0)	1 (0.1)
*SARS-CoV-2 RNA (quantitative assay), n* (%)				
High (> 1,000,000 copies/mL)	11 (45.8)	19 (61.3)	421 (61.5)	398 (59.6)
Low (500 to ≤ 1,000,000 copies/mL)	6 (25.0)	9 (29.0)	176 (25.7)	171 (25.6)
Undetectable (< 500 copies/mL)	6 (25.0)	3 (9.7)	66 (9.6)	83 (12.4)
Unknown^a^	1 (4.2)	0 (0.0)	22 (3.2)	16 (2.4)
*SARS-CoV-2 nucleocapsid antibody, n* (%)				
Positive	5 (20.8)	8 (25.8)	134 (19.6)	143 (21.4)
Negative	19 (79.2)	21 (67.7)	538 (78.5)	514 (76.9)
Unknown^a^	0 (0.0)	2 (6.5)	13 (1.9)	11 (1.6)
*SARS-CoV-2 spike protein neutralizing antibody, n* (%)				
Positive	4 (16.7)	6 (19.4)	186 (27.2)	182 (27.2)
Negative	20 (83.3)	25 (80.6)	493 (72.0)	481 (72.0)
Unknown^a^	0 (0.0)	0 (0.0)	6 (0.9)	5 (0.7)

Abbreviations: *COVID-19* coronavirus disease 2019, *MITT* modified intention-to-treat, *SARS-CoV-2* severe acute respiratory syndrome coronavirus 2

^a^Missing data, invalid sample, tests not done, or results reported as “unknown” were categorized as unknown

**Fig. 1:**
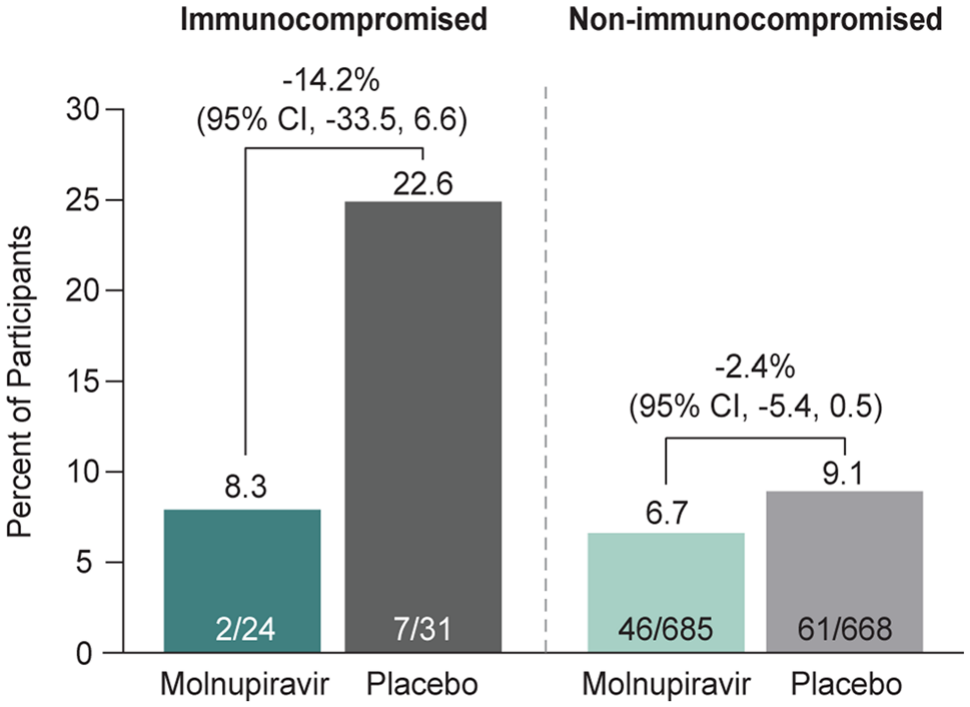
Incidence of hospitalization or death through Day 29 by immunocompromised status (MITT population). 95% CIs were based on the Miettinen and Nurminen method. Unknown survival status at Day 29 was imputed as hospitalization or death. Abbreviations: *CI* confidence interval, *MITT* modified intention-to-treat

Fewer immunocompromised participants were hospitalized or died through Day 29 in the molnupiravir group (8.3% [2/24]) compared to the placebo group (22.6% [7/31]) (difference -14.2%, 95% CI – 33.5 to 6.6) (Fig. 1). Of note, the two participants in the molnupiravir group who were hospitalized included an 81-year-old male with diffuse large B-cell lymphoma who had received cyclophosphamide, doxorubicin, vincristine, and prednisone plus rituximab 10 days prior to the first dose of study drug and was hospitalized for community-acquired pneumonia on study Day 7 and died due to multiorgan failure on study Day 26, and a 49-year-old male with active cancer (unspecified) status post-hemicolectomy who was hospitalized on study Day 8 for a perianal abscess (Supplementary Table 1). Details regarding the seven immunocompromised placebo-treated participants who were hospitalized are provided in Supplementary Table 2. In a supportive analysis, one (4.2% [1/24]) participant in the molnupiravir group and five (16.1% [5/31]) participants in the placebo group had a hospitalization or death through Day 29 considered by the investigators to be related to COVID-19.

The proportion of molnupiravir-treated participants who were hospitalized or died was similar regardless of immunocompromised status. A higher proportion of immunocompromised participants were hospitalized or died in the placebo group compared to non-immunocompromised participants (Fig. 1). A higher proportion (5.5% ([3/55]) of immunocompromised participants died versus non-immunocompromised participants (0.5% [7/1353]).

Molnupiravir was generally well-tolerated in immunocompromised participants, and the adverse event profile of molnupiravir was similar regardless of immunocompromised status (Table 3). In immunocompromised participants, there were fewer adverse events and serious adverse events in the molnupiravir group (25% [6/24] and 8.3% [2/24], respectively) compared with the placebo group (45.2% [14/31] and 19.4% [6/31], respectively), whereas in non-immunocompromised participants the adverse event profile of molnupiravir was comparable to placebo. There were three deaths in immunocompromised participants (one in the molnupiravir group and two in the placebo group) (Supplementary Tables 1 and 2) resulting from adverse events, none of which were related to study drug according to the investigators’ assessment.

**Table 3:** Adverse event summary by immunocompromised status (safety population)

	Immunocompromised				Non-Immunocompromised			
	Molnupiravir		Placebo		Molnupiravir		Placebo	
	*n*	(%)	*n*	(%)	*n*	(%)	*n*	(%)
*Participants in population*	24		31		686		670	
with one or more adverse events	6	(25.0)	14	(45.2)	210	(30.6)	217	(32.4)
with drug-related^a^ adverse events	0	(0.0)	3	(9.7)	57	(8.3)	56	(8.4)
with serious adverse events	2	(8.3)	6	(19.4)	47	(6.9)	61	(9.1)
who died	1	(4.2)	2	(6.5)	1	(0.1)	10	(1.5)
*Discontinued study drug due to*								
an adverse event	0	(0.0)	1	(3.2)	10	(1.5)	19	(2.8)
a drug-related adverse event	0	(0.0)	0	(0.0)	4	(0.6)	3	(0.4)

^a^Determined by the investigator to be related to study drug

**Fig. 2:**
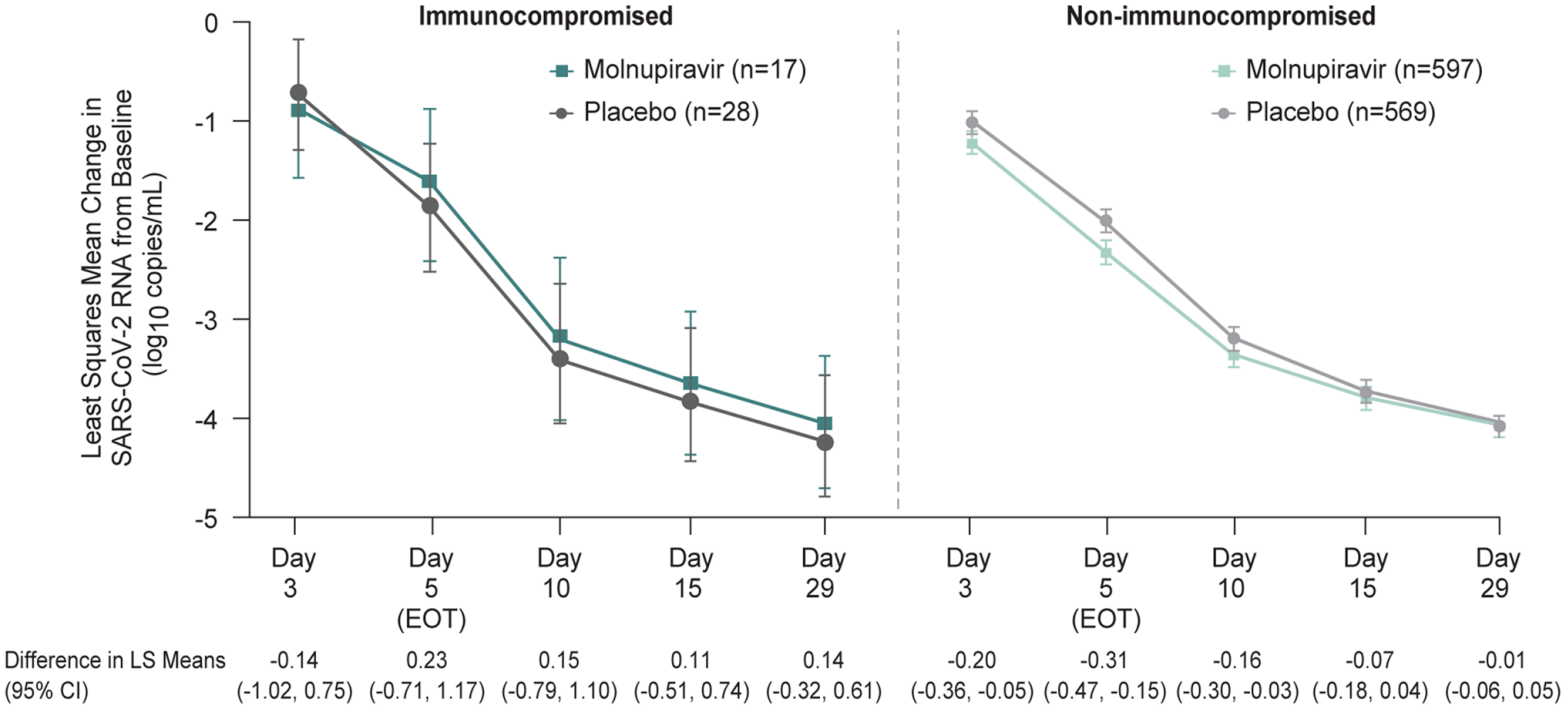
Mean change from baseline in SARS-CoV-2 RNA over time (MITT population). Error bars represent 95% CIs based on constrained longitudinal data analysis model with RNA titer as the response variable and the following variables as covariates: treatment, study visit, treatment by study visit interaction, and time from symptom onset prior to randomization (≤ 3 days vs. > 3 days from randomization strata). Analysis includes participants with baseline SARS-CoV-2 RNA titer ≥ 500 copies/mL. Abbreviations: *CI* confidence interval, *EOT* end-of-therapy, *LS* least squares, *MITT* modified intention-to-treat, *SARS-CoV-2* severe acute respiratory syndrome coronavirus 2

The mean RNA titer (log_10_ copies/mL) on Day 1 was comparable, but tended to be slightly higher among immunocompromised participants (molnupiravir 6.98 [*n* = 17] and placebo 7.07 [*n* = 28]) compared to non-immunocompromised participants (molnupiravir 6.88 [*n* = 597] and placebo 6.90 [569]) in both treatment groups. Among non-immunocompromised participants, a larger mean change from baseline in SARS-CoV-2 RNA was observed in molnupiravir-treated participants compared with placebo at all timepoints (most notable on Days 3, 5 [EOT], and 10), while the mean change was generally comparable between groups in the immunocompromised cohort (Fig. 2).

**Fig. 3:**
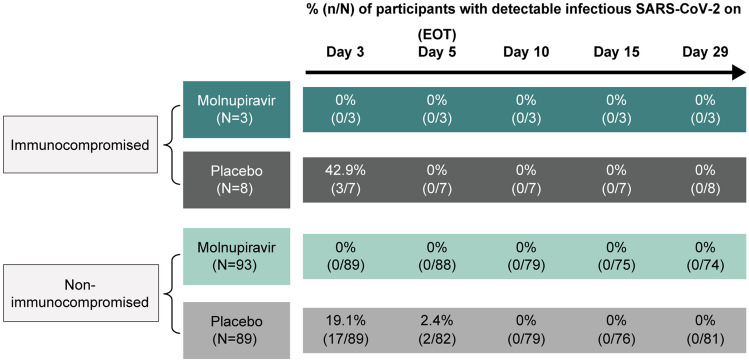
Proportion of participants with detectable infectious SARS-CoV-2 over time (MITT population). *n* = number of participants with detectable infectious titer at each timepoint among N. *N* = number of participants with positive baseline infectivity result and with available post-baseline SARS-CoV-2 RNA samples at the timepoint of analysis. Infectivity results were imputed as undetectable if the sample was not sent for infectivity testing due to SARS-CoV-2. RNA copies being lower than 10.^5^ copies/mL. Abbreviations: *EOT* end-of-therapy, *MITT* modified intention-to-treat, *SARS-CoV-2* severe acute respiratory syndrome coronavirus 2

Among participants with detectable infectious virus at baseline, no participants treated with molnupiravir had infectious virus detected at any post-baseline visit, regardless of immunocompromised status (Fig. 3). In the placebo group, 42.9% (3/7) of participants had infectious virus detected at Day 3 in the immunocompromised cohort, and 19.1% (17/89) and 2.4% (2/82) of participants had infectious virus detected at Days 3 and 5, respectively, in the non-immunocompromised cohort.

**Fig. 4:**
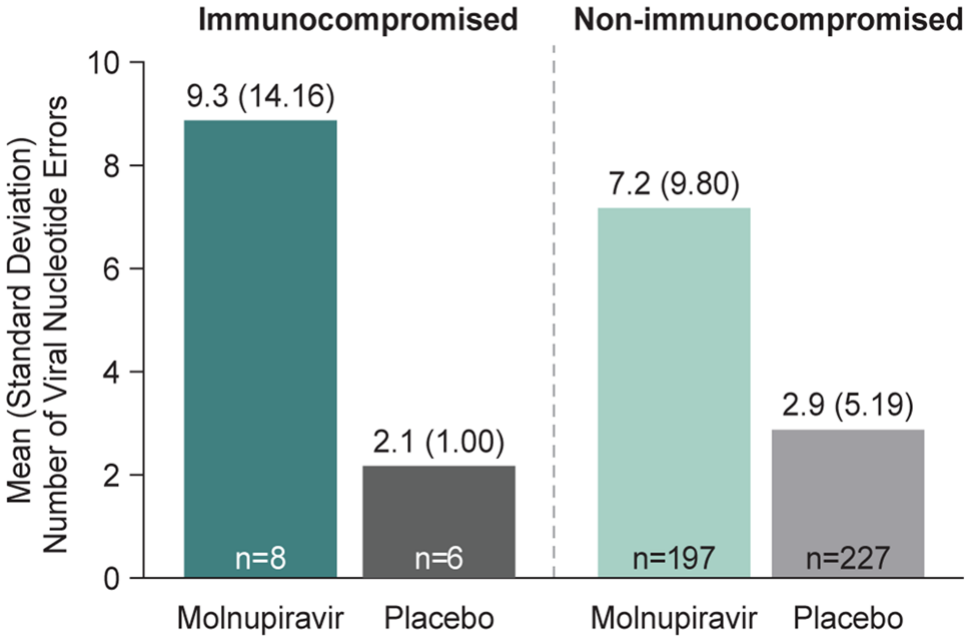
SARS-CoV-2 nucleotide error rates on Day 5 (MITT population). Viral nucleotide error rate was calculated as the number of nucleotide errors compared with the baseline sequence per 10,000 bases across the entire viral genome (30,000 bases). Abbreviations: *MITT* modified intention-to-treat, *SARS-CoV-2* severe acute respiratory syndrome coronavirus 2

The relative increase in viral nucleotide error rate in the molnupiravir group compared to placebo was generally consistent between immunocompromised and non-immunocompromised participants at Day 5 (Fig. 4). Treatment-emergent amino acid substitutions observed among molnupiravir-treated immunocompromised participants were similar to those among molnupiravir- or placebo-treated non-immunocompromised participants, with no unique protein loci substitutions observed in the viral replicase or spike sequences (Table 4). There were no treatment-emergent substitutions in viral replicase proteins NSP7, 8, 9, or 10 in immunocompromised participants.

**Table 4:** Treatment-emergent amino acid substitutions by Day 29 (MITT population)

Viral protein	Amino acid substitution	Immunocompromised		Non-immunocompromised	
		Molnupiravir (*n* = 8)	Placebo (*n* = 6)	Molnupiravir (*n* = 208)	Placebo (*n* = 234)
*Viral replicase complex*					
NSP7	Any change	0 (0.0%)	0 (0.0%)	9 (4.3%)	1 (0.4%)
NSP8	Any change	0 (0.0%)	0 (0.0%)	12 (5.8%)	3 (1.3%)
NSP9	Any change	0 (0.0%)	0 (0.0%)	12 (5.8%)	6 (2.6%)
NSP10	Any change	1 (12.5%)	0 (0.0%)	12 (5.8%)	2 (0.9%)
NSP12	Any change	3 (37.5%)	0 (0.0%)	36 (17.3%)	8 (3.4%)
	S647N/FS	1	0	1	0
	T739I	1	0	0	1
NSP13	Any change	2 (25.0%)	0 (0.0%)	35 (16.8%)	15 (6.4%)
	S259L	1	0	1	1
	A568S/V	1	0	1	0
NSP14	Any change	2 (25.0%)	0 (0.0%)	32 (15.4%)	13 (5.6%)
	A320V	1	0	1	0
Spike protein	Any change	6 (75.0%)	0 (0.0%)	95 (45.7%)	85 (36.3%)
	L5F	1	0	2	1
	G103D/C	1	0	1	0
	Y144T/FS	1	0	5	1
	D614G	1	0	0	1

Abbreviations: *MITT* modified intention-to-treat. Every participant was counted a single time for each applicable row and column. A 5% frequency cutoff was applied to identify treatment-emergent amino acid substitutions. Treatment-emergent amino acid changes were reported if detected at the same amino acid position in ≥ 2 participants (pooled molnupiravir and placebo) in post-baseline samples. Only treatment-emergent changes occurring in at least one immunocompromised participant are shown. Mixtures of amino acid changes at specific protein positions were counted only once for each participant

**Fig. 5:**
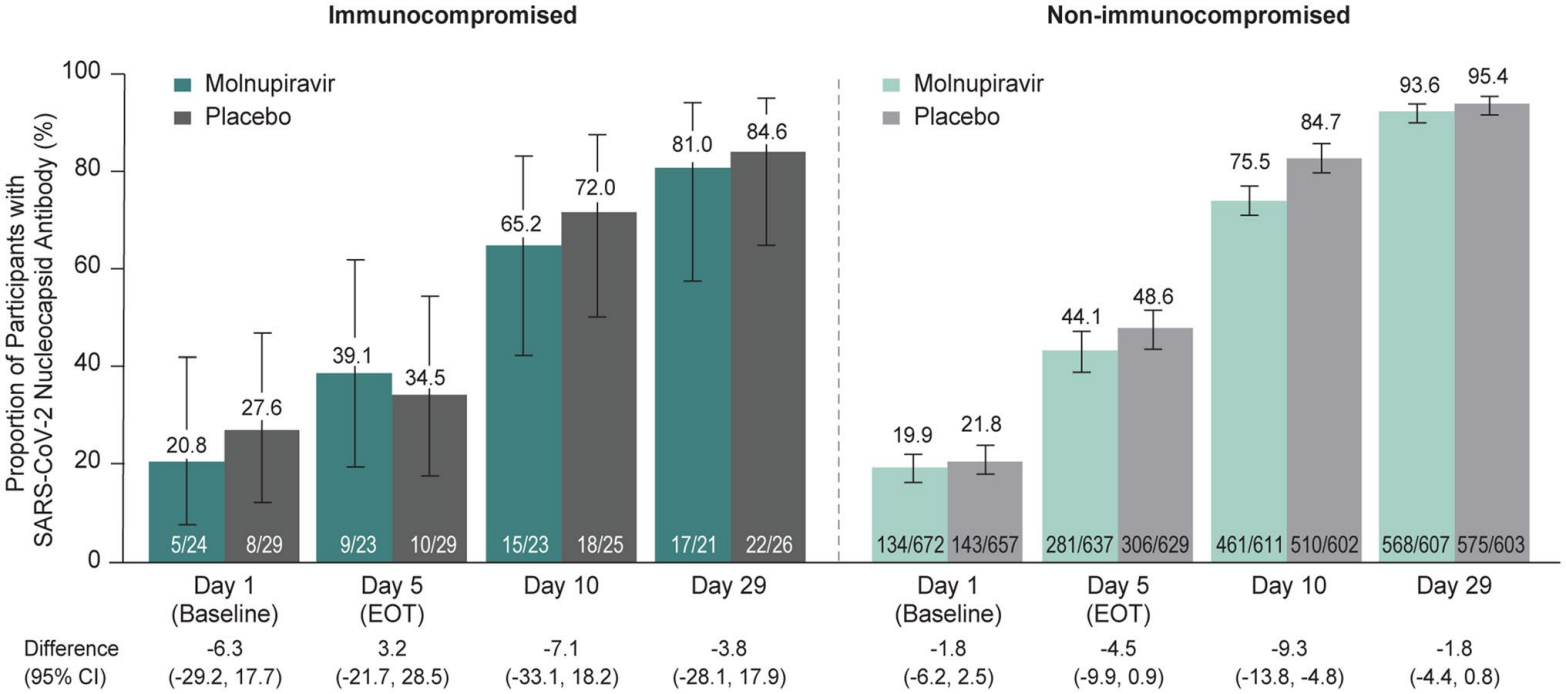
SARS-CoV-2 nucleocapsid antibody positivity over time (MITT population). *n* = number of participants with detectable SARS-CoV-2 nucleocapsid antibody at the corresponding visit; *N* = number of participants with an assay result of SARS-CoV-2 nucleocapsid antibody at the corresponding visit. 95% CI for the proportion of participants with detectable nucleocapsid antibody was based on the Clopper–Pearson method. The 95% CI for the difference in proportions was based on the Miettinen and Nurminen method stratified by randomization strata. Abbreviations: *CI* confidence interval, *EOT* end-of-therapy, *MITT* modified intention-to-treat, *SARS-CoV-2* severe acute respiratory syndrome coronavirus 2

**Fig. 6:**
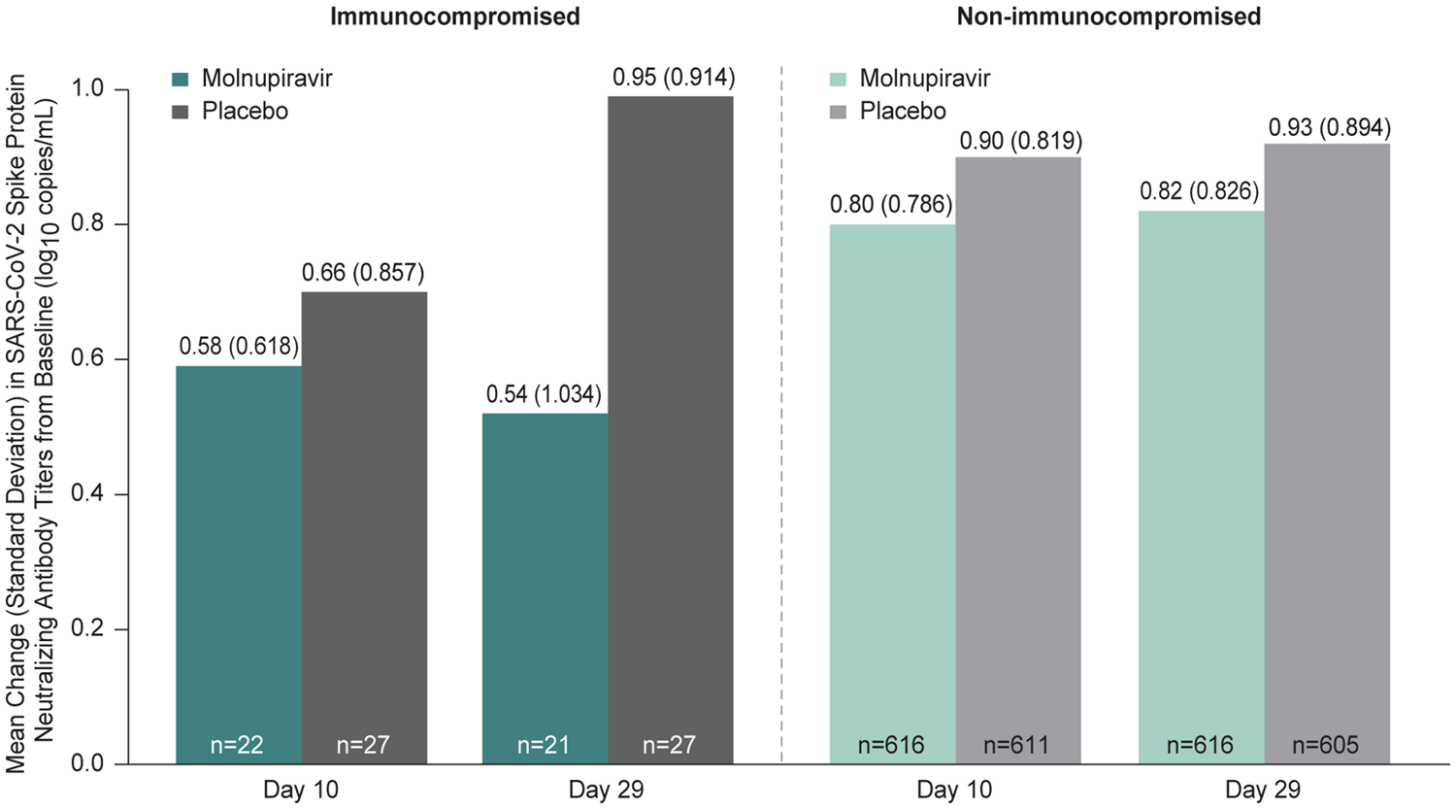
Mean change from baseline in SARS-CoV-2 spike protein neutralizing antibody titers on Days 10 and 29 (MITT population). *n* = number of participants with baseline and at least one post-baseline result at the time point assessed. The assay LLOQ was 40 copies/mL and ULOQ was 787,339 copies/mL. Post-baseline results below the LLOQ or above the ULOQ were included in the mean and mean change from baseline, with the imputed values 39 copies/mL and 787,340 copies/mL, respectively. Abbreviations: *LLOQ* lower limit of quantification, *MITT* modified intention-to-treat, *SARS-CoV-2* severe acute respiratory syndrome coronavirus 2, *ULOQ* upper limit of quantification

Nucleocapsid antibody positivity over time was generally consistent between the molnupiravir and placebo groups among both immunocompromised and non-immunocompromised participants (Fig. 5). The mean change from baseline in spike protein neutralizing antibody titers was generally comparable at Day 10 among immunocompromised participants, while a smaller mean change from baseline was observed in the molnupiravir group compared with placebo at Day 29. The mean change from baseline in spike protein neutralizing antibody titers was generally comparable between the molnupiravir and placebo groups at Days 10 and 29 among non-immunocompromised participants (Fig. 6).

## Discussion

Immunocompromised participants in phase 3 of the MOVe-OUT trial who received molnupiravir had a lower incidence of all-cause hospitalization or death without any concerning adverse events through Day 29 compared to placebo (8.3% [2/24] versus 22.6% [7/31]). Reductions in viral RNA in both treatment groups were generally consistent in immunocompromised participants, but no infectious virus was detected after baseline in any immunocompromised molnupiravir-treated participants, while infectious virus was detected post-baseline in the placebo group. Consistent with its mechanism of action, an increased viral error rate was observed in molnupiravir-treated participants, regardless of immunocompromised status. Immunocompromised participants receiving molnupiravir were not more likely to develop novel treatment-emergent amino acid substitutions in this study. Nucleocapsid antibody positivity was generally similar in both treatment groups irrespective of immunocompromised status, while a smaller mean increase from baseline in neutralizing antibody titers in molnupiravir-treated immunocompromised participants was observed at Day 29.

Delayed clearance of infectious virus in immunocompromised individuals is concerning from an infection control standpoint because of increased potential for transmission and emergence of new SARS-CoV-2 variants. Case reports have noted persistent infectious SARS-CoV-2 shedding in immunocompromised individuals [9, 23, 24]. SARS-CoV-2 evolution has also been reported in immunocompromised patients with persistent infection [23, 25–27], with the worry that monoclonal antibody therapy for COVID-19 may cause selective pressure that also contributes to the development of resistant variants [25, 26]. In this analysis, no infectious virus was detected after baseline in any immunocompromised or non-immunocompromised participants in the molnupiravir group who had infectious virus at baseline. Also, the treatment-emergent amino acid substitutions in the viral replicase and spike proteins identified in the cohort of molnupiravir-treated immunocompromised participants were not at unique locations compared to molnupiravir- or placebo-treated non-immunocompromised participants. This finding as well as the decreased infectivity suggest that it is unlikely molnupiravir will promote the development of novel SARS-CoV-2 variants.

The proportion of immunocompromised participants positive for spike protein neutralizing antibody at baseline was about 10% lower than the proportion positive in non-immunocompromised participants. In general, antibody responses were comparable in both treatment groups regardless of immunocompromised status. The smaller mean change from baseline in neutralizing antibody titers observed in immunocompromised participants in the molnupiravir group compared with the placebo group at Day 29 may have resulted from less antigenic stimulation due to earlier clearance of the virus with molnupiravir therapy.

Real-world evidence on the use of molnupiravir in immunocompromised patients is beginning to emerge in published literature [28–30]. For instance, a recent report describes clinical outcomes associated with molnupiravir therapy in immunocompromised individuals, wherein thirty-day hospitalization rates were lower with molnupiravir compared to no outpatient therapy (no molnupiravir, sotrovimab, or nirmatrelvir/ritonavir) in non-hospitalized solid organ transplant recipients with mild-to-moderate COVID-19. In this study, one intensive care unit admission, and no deaths occurred within 30 days in individuals who received molnupiravir, while there were three intensive care unit admissions and three deaths among individuals who did not receive outpatient therapy [28]. While the study population in this real-world evidence report, the majority of whom had received at least one dose of a SARS-CoV-2 vaccine and were infected during the Omicron B.1.1.529 surge, differed from the immunocompromised participants identified in phase 3 of MOVe-OUT, these real-world data align with our findings and support the use of molnupiravir in immunocompromised patients with COVID-19.

A strength of this analysis is the scope of virologic data that were evaluated in this cohort of immunocompromised participants, with analyses performed on nasopharyngeal samples that were prospectively collected at multiple timepoints in phase 3 of the MOVe-OUT trial. These data add to the very limited literature on outcomes in immunocompromised individuals treated for COVID-19 since they are usually excluded from clinical trials. Our analysis was limited by the small sample of non-hospitalized immunocompromised participants, most of whom had active cancer or well-controlled HIV; therefore, this cohort is not representative of all immunocompromised individuals, for example, patients with depleted T-cells due to uncontrolled HIV or recipients of T-cell depleting agents (i.e., antithymocyte globulin, alemtuzumab) [31–33]. Also, detailed information about cancer type and treatment was not prospectively collected in the trial database, thus the degree of immunosuppression for some participants with active cancer was not clear. Additionally, participants in MOVe-OUT were not vaccinated against SARS-CoV-2; however, recent real-world data suggest there are clinical benefits of molnupiravir therapy in vaccinated solid organ transplant recipients [28].

## Conclusions

Based on the results of this post hoc analysis in participants from phase 3 of MOVe-OUT, the use of molnupiravir appears to be effective and safe for the treatment of mild-to-moderate COVID-19 in non-hospitalized immunocompromised adults at risk for progression to severe COVID-19. There were no notable differences in virologic outcomes among molnupiravir-treated participants based on immunocompromised status, further corroborating the clinical findings in immunocompromised participants. NGS analyses taken together with infectivity data suggest that immunocompromised individuals are not more likely to develop amino acid substitutions, thereby minimizing the possibility for the development of novel SARS-CoV-2 variants following treatment with molnupiravir.

## Supplementary Information

Below is the link to the electronic supplementary material.

Supplementary file1 (DOCX 48 KB)

## Data Availability

The data sharing policy, including restrictions, of Merck & Co., Inc., Rahway, NJ, USA is available at http://engagezone.msd.com/ds_documentation.php. Requests for access to the clinical study data can be submitted through the Engage Zone site or via email to dataaccess@merck.com.
