# Supplementary material for: Molnupiravir for the treatment of COVID-19 in immunocompromised participants: efficacy, safety, and virology results from the phase 3 randomized, placebo-controlled MOVe-OUT trial
Source: Infection. 2023 Jan 17;51(5):1273–84. doi: 10.1007/s15010-022-01959-9 (PMC9844162; doi:10.1007/s15010-022-01959-9)
Supplement: Supplementary file 1 — Supplementary file1 (DOCX 48 KB) [file 15010_2022_1959_MOESM1_ESM.docx]

**SUPPLEMENT**

**Supplemental Table 1.** Immunocompromised participants who received molnupiravir and were hospitalized or died by Day 29

| Age (years) | 81 | 49 |
| --- | --- | --- |
| Sex | Male | Male |
| Country | Guatemala | Colombia |
| COVID-19 severity at baseline | Moderate | Moderate |
| Number of doses of study drug received | 9 | 10 |
| Immunocompromising condition/medication | Active cancer | Active cancer |
| Pertinent medical history | Hepatic cancer, non-Hodgkin lymphoma | Hemicolectomy, CAD, HTN, tobacco user, vasectomy |
| Prior medications | Cyclophosphamide, doxorubicin, prednisone, rituximab, vincristine | Acetylsalicylic acid, atorvastatin, *Bacillus clausii*, carvedilol, losartan, paracetamol |
| Other diagnoses or interventions while in the study | Community acquired pneumonia (Day 7) | Perianal cellulitis (Day 4), perianal abscess (Day 8), abscess drainage (Day 13) |
| Medications received through Day 29 | Acetylcysteine, budesonide, ibuprofen, levofloxacin, paracetamol, prednisone, rivaroxaban | Acetylsalicylic acid, atorvastatin, *Bacillus clausii*, bupivacaine, carvedilol, ciprofloxacin, clindamycin, diclofenac, diosmectite, enoxaparin, fentanyl, lidocaine, losartan, meropenem, metamizole, morphine, naproxen, omeprazole, paracetamol, tramadol, vancomycin |
| Hospitalization | Day 15 to Day 26 | Day 8 to Day 13 |
| Hospitalization due to COVID-19 | Yes | No |
| Death | Day 26^a^ | N/a |
| Cause of death | Multiple organ dysfunction syndrome | N/a |

Abbreviations: CAD, coronary artery disease; HTN, hypertension.

^a^Death resulted from the adverse event of community-acquired pneumonia and was deemed by investigators as unrelated to study drug.

**Supplemental Table 2.** Immunocompromised participants who received placebo and were hospitalized or died by Day 29

| Age (years) | 45 | 20 | 48 | 87 | 69 | 82 | 60 |
| --- | --- | --- | --- | --- | --- | --- | --- |
| Sex | Female | Male | Male | Female | Female | Male | Male |
| Country | Columbia | Columbia | Columbia | Mexico | Russian Federation | Ukraine | United States |
| COVID-19 severity at baseline | Moderate | Mild | Moderate | Mild | Moderate | Mild | Moderate |
| Number of doses of study drug received | 10 | 10 | 7 | 10 | 7 | 10 | 10 |
| Immunocompromising condition/medication | Active cancer | Active cancer | Prior systemic corticosteroids^a^ | Azathioprine | Leflunomide, methotrexate, prior systemic corticosteroids^a^ | Active cancer | Active cancer |
| Medical history | Hysterectomy | Left hemisphere ischemic stroke, left pneumothorax, lung metastases, monoparesis of right upper limb, osteosarcoma | CAD, diabetes mellitus, HTN, hyperuricemia | Drug hypersensitivity, HTN, Sjogren's syndrome | Abdominal compartment syndrome, arterial HTN, azotemia, cardiac ischemia, community acquired bilateral viral COVID-19-associated pneumonia, CAD, CHF, diffuse nodular goiter, dyslipidemia, hyperuricemia, joint instability, left ventricular hypertrophy, menopause, osteopenic syndrome, polyosteoarthritis, rheumatoid arthritis, thrombosis of superficial veins of the left leg, varicose veins of the lower extremities | CAD, CHF, HTN | Anxiety, arthralgia, depression, diabetes mellitus, epilepsy, hepatitis C, HTN, insomnia, osteomyelitis, peripheral neuropathy, prostate cancer, urinary retention, wound |
| Prior medications | Dalteparin, ibandronic acid, lapatinib, morphine, paracetamol, trastuzumab | Atorvastatin | Acetylsalicylic acid, allopurinol, deflazacort, piroxicam, prednisolone | Acetylsalicylic acid, azathioprine, deflazacort, fluoxetine, losartan, omeprazole | Acetylcysteine, acetylsalicylic acid, ademetionine 1,4-butanedisulfonate, leflunomide, methotrexate, methylprednisolone, metoprolol, moxonidine, omeprazole, paracetamol | Acetylsalicylic acid, ascorbic acid, melatonin, vitamin D, zinc | Acetylsalicylic acid, amlodipine, atenolol, bumetanide, carbamazepine, clonidine, diazepam, duloxetine, gabapentin, hydroxyzine, ibuprofen, lisinopril, prazosin, quetiapine, trazodone |
| Other diagnoses or interventions while in the study | Conventional oxygen (Day 8) | Conventional oxygen (Day 18), palliative care (Day 34) | Oxygen therapy (unspecified) (Day 6), oxygen by non-rebreathing mask (Day 6), ICU (Day 6) | Oxygen via nasal cannula (Day 15) | Invasive mechanical ventilation (Day 9) | Conventional oxygen (Day 2) | Invasive mechanical ventilation (Day 25) |
| Medications received through Day 29 | Ampicillin/  sulbactam, dalteparin, diosmectite, ibandronic acid, ipatinib, morphine, paracetamol, prednisone, traztuzumab | Atorvastatin | Acetylsalicylic acid, allopurinol, amoxicillin/clavulanic acid, beclomethasone, dexamethasone dihydrocodeine, diphenhydramine, enoxaparin, insulin glargine, insulin glulisine, ipratropium, losartan, omeprazole, prednisolone | Acetylsalicylic acid, azathioprine, benzonatate, deflazacort, fluoxetine, hyoscine, ipratropium, losartan, omeprazole, paracetamol | Acetylcysteine, acetylsalicylic acid, ademetionine 1,4-butanedisulfonate, leflunomide, methotrexate, methylprednisolone, metoprolol, moxonidine, omeprazole, paracetamol | Acetylsalicylic acid, ascorbic acid, melatonin, vitamin D, zinc | Acetylsalicylic acid, amlodipine, atenolol, bumetanide, carbamazepine, clonidine, diazepam, duloxetine, gabapentin, hydroxyzine, ibuprofen, lisinopril, prazosin, quetiapine, trazodone |
| Hospitalization | Day 7 to Day 12 | Day 18 to Day 41 | Day 6 to Day 21 | Day 15 to Day 20 | Day 5 to Day 11 | Day 2 to Day 20 | Day 25 to Day 32 |
| Hospitalization due to COVID-19 | Yes | No | Yes | Yes | Yes | Yes | No |
| Death | N/a | Day 41^b^ | N/a | N/a | Day 11^c^ | N/a | N/a |
| Cause of death | N/a | Cardiopulmonary arrest due to worsening lung metastases | N/a | N/a | COVID-19 infection/pneumonia and bilateral pneumonia | N/a | N/a |

Abbreviations: CAD, coronary artery disease; CHF, congestive heart failure; COVID-19, coronavirus disease 2019; HTN, hypertension; ICU, intensive care unit.

^a^For at least 4 weeks prior to the first dose of study drug.

^b^Death resulted from the adverse event of metastases to the lung and was deemed by investigators as unrelated to study drug.

^c^Death resulted from the adverse event of COVID-19/COVID-19 pneumonia and was deemed by investigators as unrelated to study drug.
